# What makes females walk at comparable speeds to males? Physical, physiological, or biomechanical factors

**DOI:** 10.1186/s40101-025-00417-3

**Published:** 2025-12-29

**Authors:** Yujin Kwon, Gwanseob Shin

**Affiliations:** 1 Department of Rehabilitation Medicine, Seoul National University Hospital, Seoul, 03080 South Korea; 2 Department of Biomedical Engineering, Ulsan National Institute of Science and Technology, Ulsan, 44919 South Korea

**Keywords:** Sex difference, Walking speed, Ankle, Range of motion, Kinematics, Biomechanics

## Abstract

**Background:**

Although males typically have longer limbs and greater muscle mass than females, previous studies have reported no significant sex differences in self-selected walking speed. This suggests that females may adopt alternative biomechanical strategies to compensate for their anatomical disadvantages. However, the specific mechanisms that enable females to achieve a walking speed comparable to males remain unclear.

**Methods:**

One hundred and fifty-one young adults (76F,75M) performed a 3-min barefoot walking at their self-selected walking speed on a 10-m walkway. During walking, spatiotemporal gait parameters, vertical ground reaction forces, and foot and ankle joint kinematics were recorded. Anthropometric measurements and body composition were also obtained to assess physical capacity. Walking speed was compared between males and females, and contributing factors to walking speed were analyzed.

**Results:**

Self-selected walking speed was not significantly different between sexes. However, when normalized by leg length, females exhibited significantly longer stride lengths and faster walking speeds than males (*p* < 0.05). Females also demonstrated greater ankle joint range of motion and walked with significantly greater ankle plantarflexion at toe-off, which likely contributed to increased forefoot pushing force. Additionally, females exhibited longer normalized stride lengths and faster stride times, resulting in faster normalized walking speeds compared to males.

**Conclusion:**

The findings suggest that females compensate for relatively shorter limb lengths and lower muscle mass by relying more on distal joint flexibility and passively generated push-off power, particularly at the ankle. Females may also adjust stride length and stride time to achieve walking speeds comparable to males despite anatomical differences. Interventions aimed at modifying push-off strategies may enhance walking efficiency and could inform the development of targeted, sex-specific gait training programs.

## Background

Human physiological and physical characteristics significantly influence how they walk, particularly how *fast* they walk. It is well established that anthropometric factors such as height and leg length affect stride length and consequently, walking speed [1, 2]. In addition, the strength and functionality of muscles spanning the lower extremity joints can affect walking patterns. For example, previous studies have shown that weakness in distal muscles (e.g., ankle plantarflexors) can diminish push-off power during walking, thereby lowering walking speed [3, 4]. As walking is an energy-demanding activity, humans naturally adopt a self-selected walking speed that minimizes metabolic cost, which is influenced by their unique physical and physiological characteristics [3]. Given these biological considerations, one might expect males and females to exhibit different walking speeds due to inherent differences in these attributes. Males who generally have longer limbs and greater muscle mass than females [5–7] are expected to generate greater forward propulsion and adopt longer stride lengths, resulting in faster walking speeds.

Contrary to these expectations, however, a number of studies investigating sex-related differences in self-selected walking speed have frequently reported no significant differences between males and females. A study that measured the preferred walking speed of 99 young adults in the USA during a 30-foot barefoot walk found average speeds of 1.34 m/s for females and 1.30 m/s for males, with no statistically significant difference [8]. Another study conducted in Canada with 154 healthy adults aged 20–75 years reported no significant sex difference in self-selected walking speed during a 6-m shod walk (females: 1.35 m/s, males: 1.34 m/s) [9]. Additionally, a study conducted in Japan found no significant difference in self-selected walking speed between females and males across young, middle, and older age groups during a 10-m barefoot walk [10]. These findings challenge the assumption that males, given their anthropometric and muscular advantages, would naturally walk faster than females. Instead, they point to the possibility that walking speed is influenced by other factors — potentially including physiological capacities or biomechanical strategies that compensate for anatomical differences. Nevertheless, the specific mechanisms by which females achieve walking speeds comparable to males remain poorly understood.

Taken together, these findings suggest that while physical and physiological differences between males and females are well acknowledged, they do not fully explain the observed similarities in self-selected walking speed. This raises an important question: What alternative mechanisms allow females to walk as fast as males despite their generally shorter limb lengths and lower muscle mass? To address this, we hypothesized that females and males would employ different biomechanical strategies to walk at comparable speeds. To test the hypothesis, we compared spatiotemporal gait parameters, vertical ground reaction forces, lower leg joint kinematics, and ankle range of motion between two sex groups to explore physical characteristics or gait patterns that may contribute to their comparable walking speeds. Understanding these mechanisms has important implications for clinical gait assessment, rehabilitation, and the development of sex-specific interventions designed to improve walking performance.

## Methods

### Participant demographics and anthropometric measurements

Individuals aged between 18 and 35 years with no current musculoskeletal problems for walking barefoot were recruited from the university community. One hundred and fifty-one participants (76F, 75M) participated and provided demographic data including biological sex assigned at birth (Table 1) after signing an informed consent form. Anthropometric variables were measured for each participant at the beginning of data collection. Body mass and body composition including muscle mass percentage, body fat percentage, and muscle circumference were measured with a bio-impedance body composition meter (Inbody 770, InBody Inc., Seoul, Korea) (Table 1). Height was measured while each participant was standing upright and barefoot on a stadiometer. Foot length was measured from the posterior heel to the tip of the longest toe, and toe length was measured from the first metatarsal head to the longest toe. Leg length was measured while the participant was lying supine on a flat massage table, from the anterior superior iliac spine to the medial malleolus bone [11]. All length measurements (leg, foot, and toe) were taken twice and averaged for each side.

**Table 1:** Demographic and anthropometric characteristics of study participants in means and standard deviations (SD)

	Female (*n* = 76)	Male (*n* = 75)	*p*-value	*t*-value
Right leg dominant (*n*)	74	65		
Age (years)	24.0 (*SD* 3.5)	24.3 (3.2)	0.627	−0.487
18–20 years (*n*)	15	13		
21–25 years (*n*)	36	39		
26–30 years (*n*)	20	20		
31–35 years (*n*)	5	3		
Height (cm)	161.0 (5.3)	174.7 (5.2)	< 0.001*	−15.982
Leg length (cm)	82.9 (3.6)	91.9 (4.3)	< 0.001*	−13.950
Foot length (cm)	23.0 (0.9)	25.6 (1.4)	< 0.001*	−13.723
Toe length (cm)	6.2 (0.5)	6.8 (0.5)	< 0.001*	−6.913
Mass (kg)	54.3 (7.5)	72.6 (8.6)	< 0.001*	−13.916
BMI (kg/m^2^)	21.0 (2.8)	23.8 (2.7)	< 0.001*	−6.369
Muscle mass percentage (%)	37.6 (3.4)	45.1 (3.6)	< 0.001*	−13.154
Body fat percentage (%)	29.8 (6.2)	19.7 (6.1)	< 0.001*	10.048
Thigh muscle circumference (cm)	41.9 (2.3)	48.6 (2.8)	< 0.001*	−16.226
Active DF ROM (*n* = 23; 12F, 11M)	19.2 (6.8)	20.7 (5.4)	0.563	−0.588
Active PF ROM (*n* = 23; 12F, 11M)	55.8 (6.2)	42.0 (11.5)	0.002*	3.628

Asterisks (*) indicate a significant difference between females and males.

*DF ROM* dorsiflexion range of motion.

### Gait data collection

Gait data collection was conducted in a laboratory setting using a 10-m straight walkway. Participants walked barefoot at their self-selected, preferred walking speed for 3 min continuously. At each end of the walkway, they turned and continued walking without pausing. A 1.5-m plantar pressure distribution measurement system (Zebris FDM, Zebris Medical GmbH, Germany) was installed in the middle of the walkway, which measured pressure distribution and the vertical component of ground reaction forces. The entire walkway was covered with black adhesive vinyl sheet to conceal the location of the measurement system. Plantar pressure and force distributions were recorded at 100 Hz as participants walked over the platform.

Lower extremity segment angle and angular acceleration data were collected by inertial measurement units (IMUs) (Xsens MTw, Xsens, the Netherlands) at the sampling rate of 100 Hz. Two IMU sensors were attached to the dorsal surfaces of the feet and the front side of the shanks, and a single IMU was attached to the pelvis at the lumbosacral joint level. Segment angles during gait were calculated relative to upright standing reference angles, which were defined as neutral and obtained at the beginning of the session.

### Data processing and analysis

Given that the study participants walked at 1.15 m/s (Table 2), the number of measurable steps used to calculate spatiotemporal gait parameters ranged from approximately 21 to 42. Spatiotemporal parameters, including step length and stride time were analyzed to examine the effects of gait characteristics on walking speed. To minimize confounding effects and enable meaningful comparisons among individuals with different anthropometric characteristics, all gait parameters were normalized to each participant’s leg length [12]. Additional variables were obtained from the plantar pressure measurement system to investigate the effects of the forward propulsion strategy of plantar forces on walking speed. These included the following: stance phase (% stride time, duration of ground contact), divided into three sub-phases — loading response (initial contact to contralateral toe-off), mid-stance (contralateral toe-off phase), and pre-swing (contralateral initial contact to ipsilateral toe-off); double stance phase (% stride time, ground contact time for both feet); contact time of heel (0~33% of foot length), midfoot (33~67%), and forefoot (67~100%) (% stance phase) [13]; length of gait line (length of center of pressure path); max gait line velocity (maximum center of pressure velocity); time change from heel to forefoot (duration from heel contact to forefoot contact); the first and second peak values of the vertical ground reaction force (vGRF); and the maximum vGRF of the forefoot, midfoot, and heel. All force values (N) were normalized to body weight (BW).

**Fig. 1:**
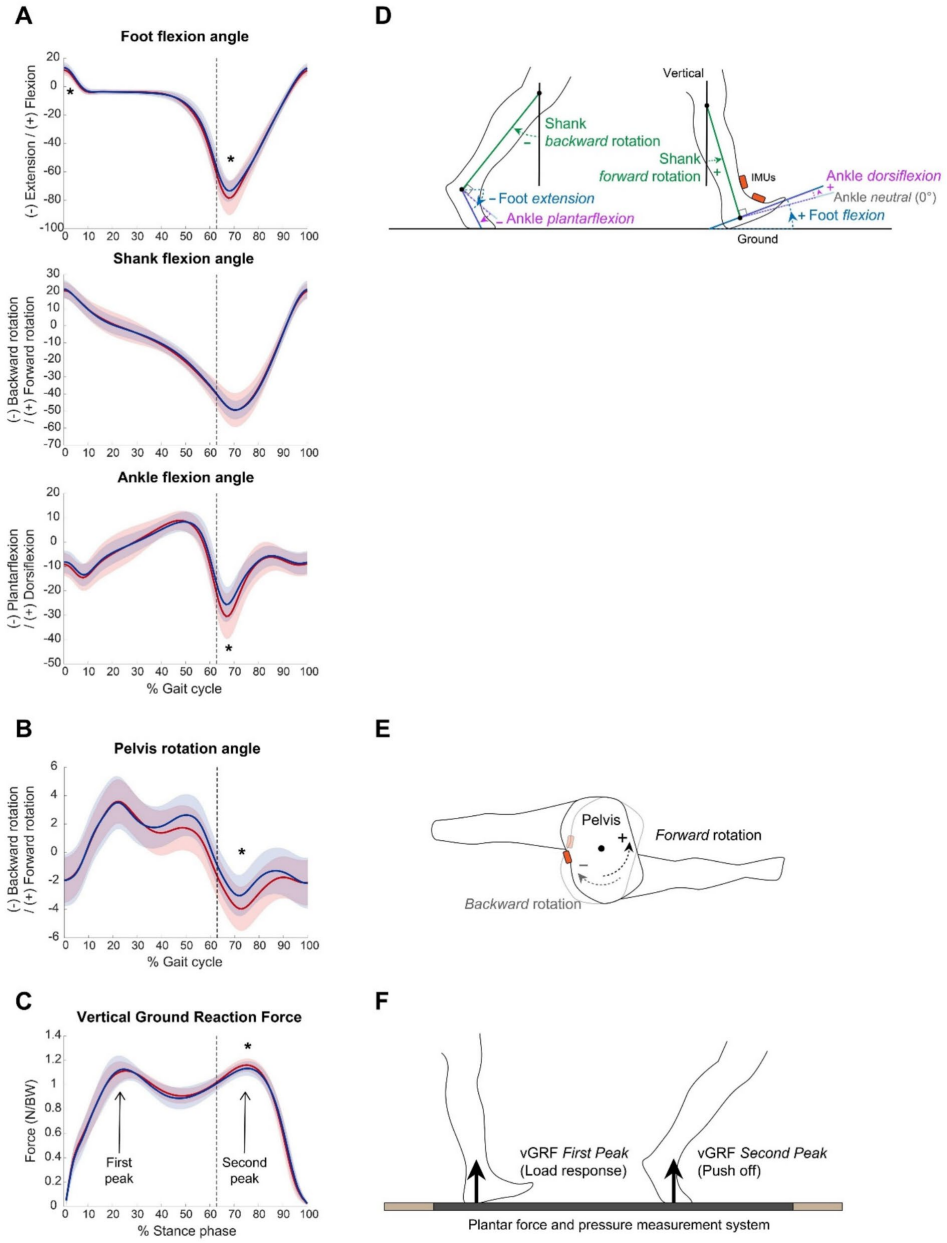
Segment and joint kinematics (**A**, **B**) and vertical ground reaction force (**C**). Shaded areas in the graphs represent ±1 standard deviation across participants. The vertical dashed line represents averaged toe-off time. Axes of rotation and calculation of variables of interest are described (**D**, **E**, **F**). Asterisks (*) indicate a significant difference in the peak value between females (red) and males (blue)

The IMU signals from the first and last steps at both ends of the walkway prior to turning were excluded from data analysis to eliminate acceleration and deceleration phases. The segment rotation and acceleration signals from the remaining steps were smoothed using the 4^th^ order Butterworth filter with a cut-off frequency of 20 Hz. The instants of heel contact and toe-off were identified from the sagittal plane rotation data of the foot segment [14]. Foot and ankle joint kinematic variables were calculated from the IMU sensors to investigate the effects of heel strike or push-off strategies on walking speed. The variables included sagittal plane angles of the foot and shank, ankle flexion angles at heel strike and toe-off, and their peak values during the stance and swing phases (Fig. 1D). Peak pelvis forward and backward rotation angles in the transverse plane were calculated from the pelvis IMU (Fig. 1E). Although the plantar pressure distribution measurement system and IMU sensors were not synchronized, data were recorded over the same path and duration. All kinematic variables were averaged across all valid steps for each participant. The data from the dominant limb were used for data analysis.

The calculated variables were compared between the two sex groups. The effects of sex on the anthropometric variables, gait parameters, vertical ground reaction forces, and kinematic variables were evaluated using a two-sample *t*-test. Correlation analysis was conducted when necessary for further examination. All statistical analyses were performed using R (Rstudio, Boston, MA, USA) with a significance level of 0.05.

## Results

### Demographic and anthropometric characteristics of study participants

The mean age of female and male participants was 24.0 ± 3.5 years and 24.3 ± 3.2 years, respectively (Table 1). A majority of participants were right-leg dominant (defined as the leg used to kick a ball), with 74 out of 76 females (97.4%) and 65 out of 75 males (86.7%) reporting right-leg dominance. Males had significantly greater height, mass, body mass index (BMI), and thigh muscle circumference but lower body fat percentage compared to females (*p* < 0.001). The lengths of the leg, foot, and toe were significantly longer for males than females (*p* < 0.001). When normalized by height, males exhibited significantly longer foot length (*p* = 0.001) and leg length (*p* < 0.001) than females.

### Gait parameters and vertical ground reaction forces

Self-selected walking speed was not significantly different between sexes, while the speed normalized by leg length was faster for females than for males (*p* < 0.001) (Table 2). Males exhibited a longer stride length and stride time than females (*p* = 0.001). However, after normalization, females walked with a significantly longer stride length than males (*p* = 0.002). Females exhibited a greater normalized second peak of the vGRF (*p* = 0.002) and maximum forefoot force (*p* < 0.001), while males walked with a greater midfoot force compared to females (*p* = 0.031).

**Table 2:** Gait parameters and vertical ground reaction forces

	Female (*n* = 76)	Male (*n* = 75)	*p*-value	*t*-value
*Gait parameters*				
Walking speed (m/s)	1.15 (*SD* 0.13)	1.17 (0.20)	0.430	−0.791
Walking speed (/leg length)	1.39 (0.16)	1.28 (0.21)	< 0.001*	3.750
Stride length (m)	1.20 (0.10)	1.27 (0.14)	0.001*	−3.319
Stride length (/leg length)	1.45 (0.12)	1.39 (0.14)	0.002*	3.229
Stride time	1.05 (0.07)	1.10 (0.10)	0.001*	−3.456
Stance phase (%)	62.8 (1.2)	62.8 (1.6)	0.955	−0.057
Loading response (%Stance)	20.0 (1.9)	20.2 (2.8)	0.688	0.403
Mid-stance (%Stance)	59.4 (3.0)	58.0 (6.0)	0.058	1.908
Pre-swing (%Stance)	20.5 (1.5)	20.3 (2.9)	0.565	0.577
Double stance phase (%)	25.5 (2.4)	25.9 (3.4)	0.368	−0.903
Contact time forefoot (%Stance)	95.0 (1.8)	83.3 (5.0)	0.349	0.939
Contact time midfoot (%Stance)	94.8 (1.6)	83.1 (4.2)	0.824	0.223
Contact time heel (%Stance)	65.6 (4.8)	66.6 (6.5)	0.302	−1.036
Length of gait line (mm)	207.9 (14.3)	232.9 (11.3)	< 0.001*	−11.504
Length of gait line (%Foot length)	90.5 (5.7)	91.3 (3.3)	0.317	−1.003
Max gait line velocity (mm/s)	206.6 (69.7)	226.9 (71.3)	0.089	−1.714
Time change heel to forefoot (s)	0.260 (0.043)	0.276 (0.059)	0.071	−1.817
Time change heel to forefoot (%Stance)	61.7 (8.8)	61.9 (9.3)	0.909	−0.114
*Vertical ground reaction force*				
vGRF first peak (BW)	1.13 (0.07)	1.15 (0.10)	0.295	−1.050
vGRF second peak (BW)	1.18 (0.07)	1.14 (0.06)	0.002*	3.232
Maximum force - forefoot (BW)	1.16 (0.06)	1.12 (0.06)	< 0.001*	3.752
Maximum force - midfoot (BW)	0.22 (0.07)	0.25 (0.09)	0.031*	−2.184
Maximum force - heel (BW)	0.78 (0.07)	0.77 (0.09)	0.309	1.022

Asterisks (*) indicate a significant difference between females and males

### Foot, ankle, and pelvis kinematics

Foot, shank, ankle, and pelvis kinematics are presented in Fig. 1 and Table 3. The percentage of the stance phase within a gait cycle was not different between females and males (*p* = 0.955). At heel strike, the foot flexion angle was significantly larger for males than females (*p* = 0.028), while the shank and ankle flexion angles were not. The maximum ankle dorsiflexion angle during the late stance phase (push-off) was not significantly different between sexes (*p* = 0.407). At toe-off, the foot extension (*p* = 0.002) and ankle plantarflexion (*p* = 0.003) angles were significantly larger for females than males. The difference in the maximum foot extension and ankle plantarflexion angles increased in the swing phase (*p* < 0.001). The pelvis backward rotation was significantly larger for females than males (*p* = 0.008), while the forward rotation was not significantly different between sexes.

**Table 3:** Foot, ankle, and pelvis kinematics data

	Female (*n* = 76)	Male (*n* = 75)	*p*-value	*t*-value
Foot rotation (°)	6.28 (4.18)	8.93 (5.47)	0.001*	−3.331
*At heel strike*				
Foot flexion angle (°)	12.0 (3.6)	13.3 (3.7)	0.028*	−2.214
Shank forward flexion angle (°)	21.2 (4.1)	21.5 (5.2)	0.642	−0.466
Ankle plantarflexion angle (°)	−9.2 (4.6)	−8.2 (4.6)	0.208	1.265
*Stance phase*				
Max ankle dorsiflexion angle (°)	9.7 (3.7)	9.1 (4.3)	0.407	0.831
Max pelvis forward rotation (°)	3.7 (1.6)	3.9 (1.7)	0.442	−0.772
*At toe-off*				
Foot extension angle (°)	−62.0 (7.9)	−57.9 (8.2)	0.002*	3.184
Shank flexion angle (°)	−40.9 (6.2)	−40.1 (4.9)	0.418	0.813
Ankle plantarflexion angle (°)	−21.2 (7.2)	−17.7 (6.6)	0.003*	3.071
*Swing phase*				
Max foot extension angle (°)	−81.3 (7.9)	−75.7 (7.2)	< 0.001*	4.557
Max shank backward flexion angle (°)	−51.0 (6.3)	−50.0 (5.3)	0.292	1.057
Max ankle plantarflexion angle (°)	−30.2 (7.7)	−25.6 (7.0)	< 0.001*	3.842
Max pelvis backward rotation (°)	−4.1 (1.6)	−3.4 (1.5)	0.008*	2.692

Asterisks (*) indicate a significant difference between females and males

## Discussion

This study was initiated to better understand the mechanisms that enable females to walk as fast as males. Consistent with previous research, the self-selected walking speed of our young female participants did not differ from that of young male participants [8–10, 15]. However, when walking speed was normalized by leg length, females walked 8.6% faster than males. Further analysis of joint kinematics and plantar pressure data suggests that greater ankle plantarflexion at toe-off enabled females to extend the stride and generate increased forefoot pushing force. This contributed to longer normalized stride lengths and faster walking speeds compared to male participants.

In our study, females pushed off the ground with larger foot extension and ankle plantarflexion angles than males by 3.4~4.6°, and it might be related to the sex difference in ankle joint range of motion. To evaluate the effect of ankle joint flexibility on walking strategy, we measured the active ankle range of motion in the last 23 participants (out of 151) [16] and found that females (*n* = 12) demonstrated a significantly greater plantarflexion range of motion (55.8°) compared to males (*n* = 11, 42.0°, *p* = 0.002) (Table 1), consistent with the findings in previous research [17–19]. The greater range of ankle plantarflexion was accompanied by greater backward pelvis rotation, which may have allowed females to maintain ground contact with the ground for a longer duration. This, in turn, likely contributed to their longer normalized stride length compared to males. The supposition is supported by significant correlations between active ankle plantarflexion range of motion and foot extension angle at toe-off (*r* = 0.582, *p* = 0.004) and during the swing phase (*r* = 0.620, *p* = 0.002). These findings suggest that our female participants might have leveraged their greater ankle joint flexibility to achieve walking speeds comparable to that of male participants.

The greater ankle plantarflexion at toe-off might also be attributable to the significantly greater vertical ground reaction force during push-off, accompanied by larger maximum forefoot force. Greater ankle plantarflexion with the toes in contact with the ground could lead to greater hallux dorsiflexion and a flattened medial arch of the foot. The passive tension resulting from hallux dorsiflexion and the flattened medial arch could add push-off forces, a phenomenon known as the windlass mechanism [20–22]. The additional force, generated by the resistance of stretched tissues during hallux dorsiflexion while pressing against the ground, may have contributed to the greater vertical ground reaction force from the forefoot during push-off. The combination of greater ankle plantarflexion angle and vertical ground reaction force is associated with higher ankle plantarflexor moments, which are known contributors to propulsive force and, consequently, walking speed [23, 24]. Supporting this interpretation, we observed significant correlations between ankle plantarflexion angle at toe-off and walking speed (*r* = 0.211, *p* = 0.010), as well as between the second peak of the vertical ground reaction force during push-off (immediately before toe-off) and walking speed (*r* = 0.216, *p* = 0.008).

While females exhibited longer stride length relative to leg length, their faster stride time likely also contributed to their comparable walking speed to males. Walking speed is determined by both stride length (a spatial parameter) and stride time (a temporal parameter). In our study, female participants demonstrated *faster* stride time, which may represent a compensatory strategy for the absolute shorter stride length imposed by their anatomical disadvantages compared to males. Furthermore, females exhibited relatively *longer* stride length than males of the same leg length. Together, the combination of faster stride time and longer normalized stride length resulted in *faster* normalized walking speed in females. These findings collectively provide important insight into sex-specific locomotor strategies adopted despite anatomical differences, with females appearing to rely more heavily on distal joint mechanisms to adjust gait patterns and enhance walking speed.

Consistent with previous studies [5–7], our male participants had higher BMI and thigh muscle circumference, along with lower body fat percentages than female participants. This suggests that males possessed greater muscle mass than females although they were of similar height. Particularly, our finding that females demonstrated greater ankle plantarflexion at toe-off indicates that they might rely more on the passively generated push-off power than male participants to compensate for their relatively lower muscle power. This is supported by previous evidence showing that females walked with significantly larger ankle plantarflexion angles and moments during push-off compared to males, despite no significant sex differences in hip flexion or knee extension moments [8]. Similarly, Chiu and Wang (2007) [25] reported that females walked with greater ankle joint motion, increased shank muscle activity, and higher vertical ground reaction forces than males. Collectively, these findings suggest that females may leverage their greater ankle joint flexibility and rely more on distal muscles during push-off, whereas males may rely more on proximal joints to achieve comparable forward propulsion. This interpretation, however, requires further validation with muscle activity and joint kinetics data. Future studies are also warranted to investigate the physiological implications of sex-specific walking strategies including their potential benefits and drawbacks to guide the development of targeted and effective gait interventions.

There are some limitations in this study. First, this study focused on gait patterns of healthy young adults aged between 18 and 35 years, with a relatively narrow range of age (mean: 24.0 years, range: 18 to 32 years), limiting the ability to assess age-related effects. Future studies should include participants over 35 years to examine how age influences physical capacity and gait patterns. Second, we used a plantar pressure and force measurement system that measures only the vertical component of ground reaction forces. To precisely analyze the effect of push-off strategy on the mechanism of forward propulsion and walking efficiency, measurement of horizontal ground reaction force would also be necessary [26]. Future studies should incorporate a broader range of biomechanical measures to better understand complex push-off mechanisms. Additionally, active ankle PF ROM was measured only in the last 23 participants. However, we expect similar results for the remaining participants, given the strong *p*-value (Table 1) and the consistency of our findings with previous research [27–29]. Lastly, our study recruited only Korean participants, which may limit the generalizability of the findings. Although gait parameters were normalized to leg length to account for anatomical differences across populations, the results should be interpreted with caution.

## Conclusion

In conclusion, females could achieve comparable walking speeds through biomechanical adaptations, particularly greater ankle plantarflexion at toe-off — a strategy likely linked to enhanced ankle joint flexibility. These adaptations may reflect a compensatory strategy to offset relatively shorter limb length and lower muscle mass, with females relying more on distal joint motion and passive force generation during walking. Our findings underscore the critical role of ankle joint flexibility in influencing ankle kinematics and walking performance. Looking forward, a deeper understanding of how ankle mobility affects walking speed — how females employ different strategies at toe-off to achieve walking speeds comparable to males — can inform physical therapy and clinical practice. For example, clinicians may consider prescribing footwear with lower bending stiffness at metatarsophalangeal or ankle joints to facilitate greater hallux and ankle dorsiflexion, potentially enhancing walking speed [18, 30]. Additionally, gait training that focuses on increasing ankle plantarflexion and forefoot pushing force at toe-off may improve walking efficiency and performance, particularly for females.

## Data Availability

No datasets were generated or analysed during the current study.

## Abbreviations

DF: Dorsiflexion
PF: Plantarflexion
ROM: Range of motion
BMI: Body mass index
vGRF: Vertical ground reaction force
IMU: Inertial measurement unit
BW: Body weight
